# Coarse-grained (hybrid) integrative modeling of biomolecular interactions

**DOI:** 10.1016/j.csbj.2020.05.002

**Published:** 2020-05-15

**Authors:** Jorge Roel-Touris, Alexandre M.J.J. Bonvin

**Affiliations:** Bijvoet Centre for Biomolecular Research, Faculty of Science – Chemistry, Utrecht University, Utrecht 3584CH, the Netherlands

**Keywords:** Complexes, Docking, Molecular representations, Force field, Software

## Abstract

The computational modeling field has vastly evolved over the past decades. The early developments of simplified protein systems represented a stepping stone towards establishing more efficient approaches to sample intricated conformational landscapes. Downscaling the level of resolution of biomolecules to coarser representations allows for studying protein structure, dynamics and interactions that are not accessible by classical atomistic approaches. The combination of different resolutions, namely hybrid modeling, has also been proved as an alternative when mixed levels of details are required. In this review, we provide an overview of coarse-grained/hybrid models focusing on their applicability in the modeling of biomolecular interactions. We give a detailed list of ready-to-use modeling software for studying biomolecular interactions allowing various levels of coarse-graining and provide examples of complexes determined by integrative coarse-grained/hybrid approaches in combination with experimental information.

## Graphical abstract

**Figure:**
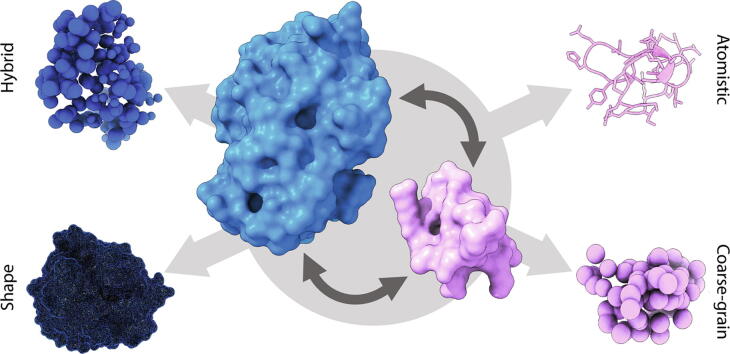

## 1 Introduction

The chemistry that supports life is extremely sophisticated. Despite advances over the past decades, the scientific community still lacks fundamental knowledge to fully understand the biology behind the cell at atomic level. We know that basic subunit atoms (i.e. carbon, oxygen, hydrogen and nitrogen) can combine and form complex molecules such as lipids, carbohydrates, nucleic acids and proteins. At the same time, these biomolecules associate and create more intricated assemblies that adopt specific three-dimensional (3D) structures, essential for their biological functions. Their interactions mediate a wide range of biological functions such as for example signal transduction, molecular recognition or transport. Indeed, roughly 80% of the proteins might function upon association with other biomolecules [1]. It is therefore of great importance to understand how these macromolecules interact. Next to experimental methods, complementary computational approaches have been develop with the so-called integrative modeling emerging as the most promising strategy [2]. In short, integrative modeling aims at obtaining structural insights into a given system under study that cannot be revealed by a single approach alone. To do so, it combines data from multiple information sources (e.g. nuclear magnetic resonance (NMR) spectroscopy, cryo-electron microscopy (cryo-EM), mass spectrometry (MS), small angle x-ray scattering (SAXS), bioinformatics analysis…) [3] into computational approaches to model the assemblies. Integrative modelling has been extensively used to model increasingly larger systems in the recent past [4]. In this sense, we are probably closer than ever to construct a predictive model of an entire cell [5].

Classical atomistic computational modeling of interactions remains inefficient for many molecular assemblies. Larger systems often require longer simulations and their complex conformational landscapes cannot be efficiently and thoroughly sampled by atomistic approaches. The simplification of large systems to coarser representations offers a valuable approach to alleviate those limitations. There is already a huge body of literature on this topic and, in the present work, we do not aspire to give the most comprehensive review covering all possible contributions, but will focus on the modeling of biomolecular interactions. i.e. complexes, involving proteins, peptides and nucleic acids (DNA and RNA). The remaining of the text is organized as follows: We first start with a brief historical overview of the development of coarse-graining. We then describe several representative designs of simplified systems and parametrization strategies and discuss how these can be implemented into the modeling of biomolecular complexes, both for the generation of possible conformations (sampling) and the discrimination between native and non-native models (scoring). Finally, we provide an overview of currently available software that support coarse-grained modeling of biomolecular complexes and highlight several representative applications.

## 2 Historical perspective

The structural characterization of lysozyme in 1967 [6] spurred Arieh Warshel to study enzymatic reaction mechanisms. His developments in this field under the supervision of Martin Karplus, inaugurated the now well-established quantum mechanics/molecular mechanics (QM/MM) methods [7]. In parallel, Michael Levitt, a PhD student at the Medical Research Council at that time, was making significant advances for studying molecular conformations by computational approaches: Together with Shneior Lifson in 1972 at the Weizmann Institute in Israel, Levitt and Warshel started working on a simplified representation of a protein, where spheres would represent amino acids. In fact, this project, later on in 1975, turned out in the very first computer simulation of a protein system (pancreatic trypsin inhibitor) using a coarse-grained model [8]. These simulations suggested that the protein folding process has a relatively small number of conformations, and challenged the so-called “Levinthal paradox” [9]. In this work, each residue was represented by only two beads: The Cα atom and the centroid of the side chain. Non-bonded interactions were assumed to occur only between side chains. By doing so, only torsion angles between 4 consecutive Cα atoms were considered, considerably reducing the conformational space (one degree of freedom per residue). For all these premature findings Karplus, Levitt and Warshel were awarded with the Nobel Prize in Chemistry in 2013.

**Fig. 1:**
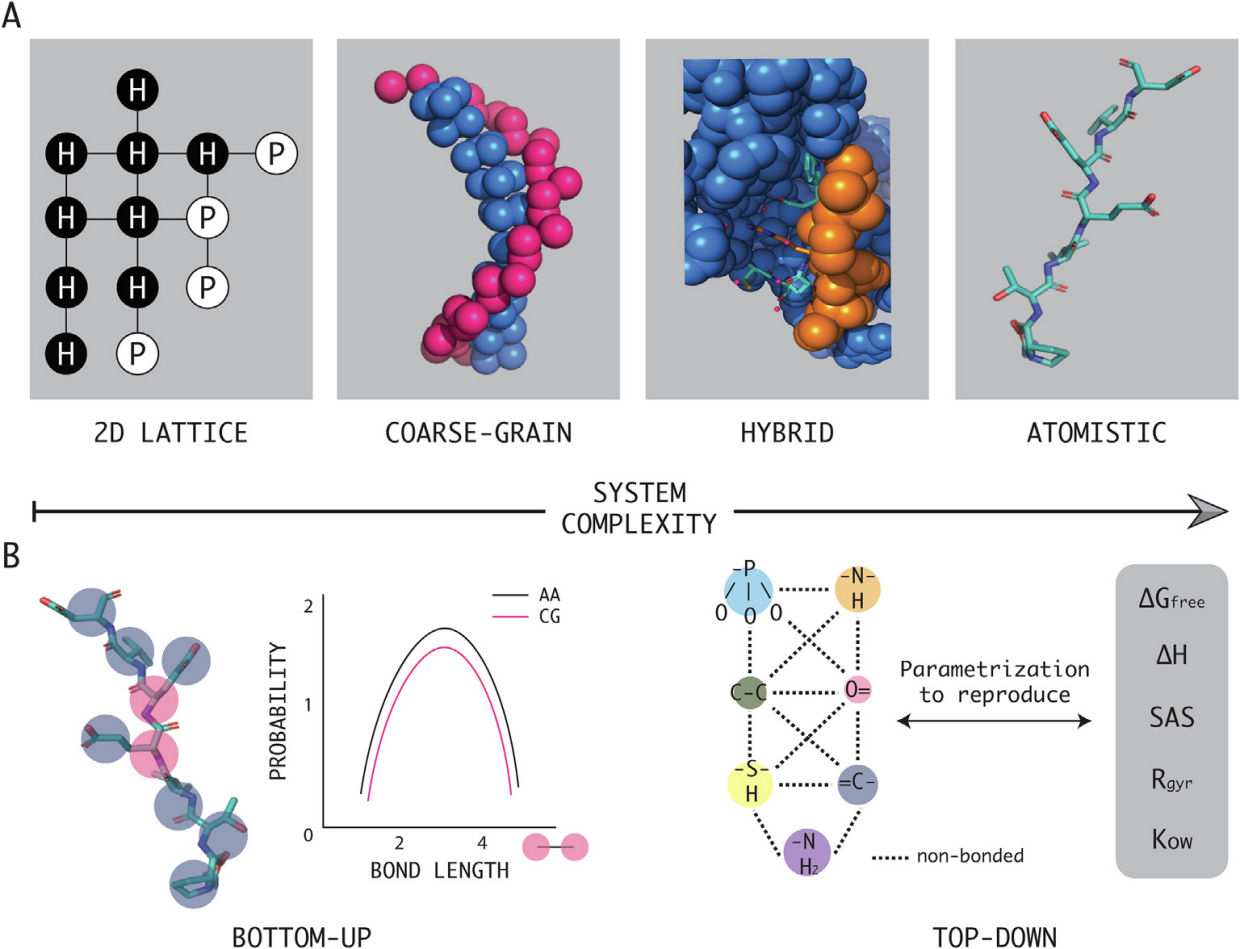
Examples of various coarse-grained models. (A) The panels from left to right illustrate the increase in the complexity of the system (i.e. decreased coarse-graining): A 2-D lattice representation of a HP model, a coarse-grained (4:1 mapping) of a dsDNA molecule, a hybrid representation of a protein–protein interface (AA/CG) and an atomistic model of a peptide. (B) The two traditional parametrization strategies. *Bottom-up*: Bond-lengths are parametrized by mapping to distributions of reference atomistic simulations. *Top-down*: Models are designed to match specific properties (e.g. thermodynamic quantities) of the system.

**Fig. 2:**
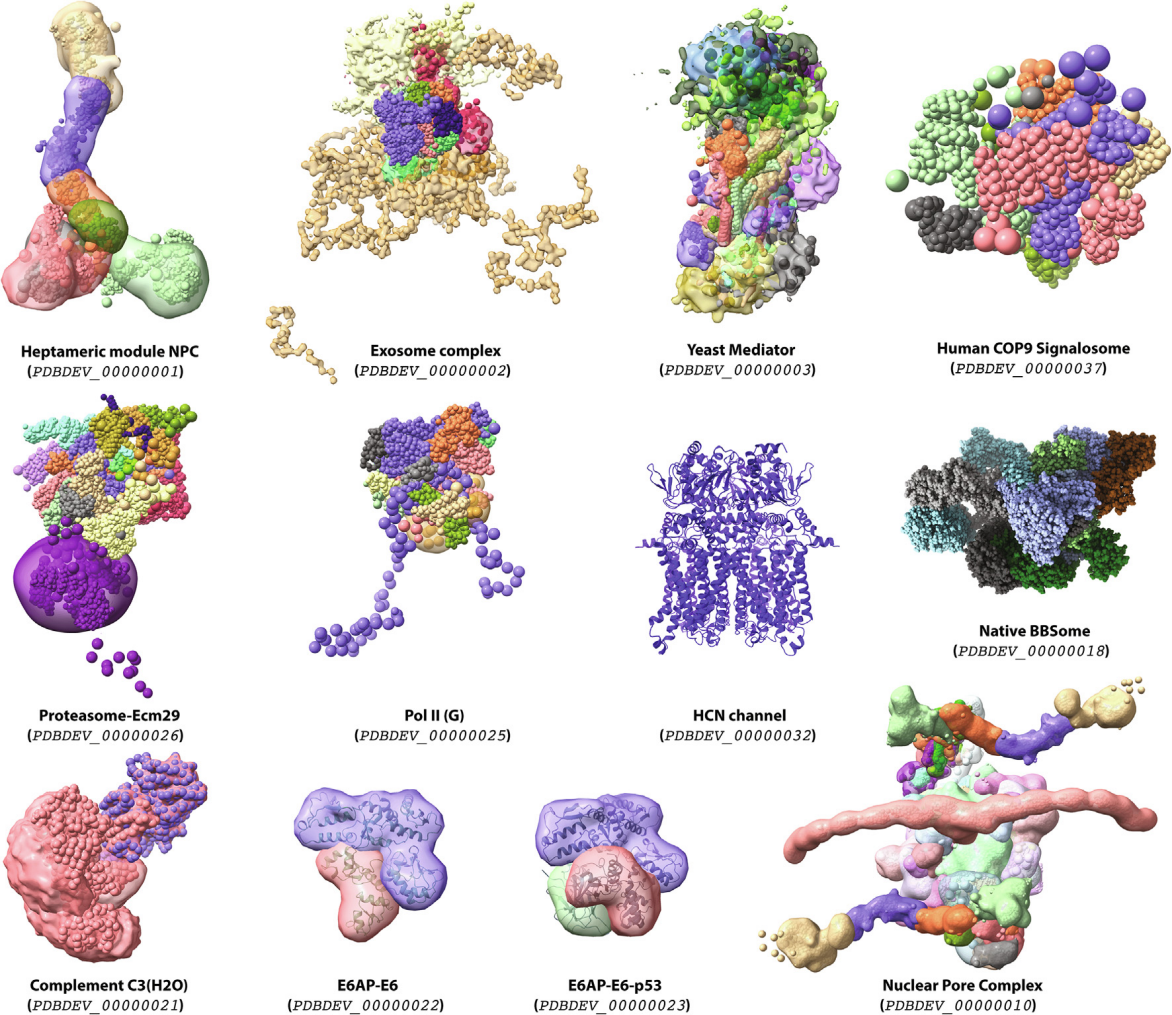
Examples of integrative structures determined by partial/full coarse-grained/hybrid computational approaches as archived in the PDB-dev database [120], [121] (pdb-dev.wwpdb.org). Pictures were generated with ChimeraX [136]. The experimental information used for the modeling (if included) has been omitted for visualization purposes. Models can be directly opened in ChimeraX from the command line as: *open [model_number] from pdbdev ignoreCache true.*

In 1975, Chothia and Janin established the structural basis of the hydrophobic effect as fundamental to the stabilization of protein association [10]. All these pioneering findings were used as a basis for the first computational analysis of a protein–protein complex: In 1978, Wodak and Janin studied the association of BPTI and trypsin using a coarse-grained representation of the system [11]. They used a combination of a simple averaged potential energy function including non-bonded (van der Waals) and residue-solvent interactions. Whilst encouraging, this early model totally neglected electrostatic interactions and was thus unable to describe hydrogen bonds and salt bridges, which, later on in 1984, were suggested to provide the specificity of the association [12]. In spite of the incompleteness of this work, they shed light on the idea that a simplified protein model could be an effective alternative to screen a relatively large number of possible interfaces, which constituted the first coarse-grained docking simulation. Ever since, coarse-grained/hybrid modeling approaches have gained importance in the computational structural biology field [13] and have become central in the study of folding, dynamics and association mechanisms of biomolecules.

## 3 Coarse-grained/Hybrid modeling of biomolecular interactions

In this section, we will focus on macromolecular docking approaches allowing some level of coarse-grained/hybrid representations for the modeling of interactions. These usually include two different steps: The generation of possible complex conformations, referred to as sampling, and the discrimination between biologically and non-biologically relevant models referred to as scoring. The latter might also be an integral part of the sampling process, especially when experimental or predicted information is included to bias the sampling (e.g. restraints-driven sampling). We first describe various strategies to simplify the representation of polypeptides and nucleic acids and discuss existing parametrization strategies and force fields. We then focus on how coarse-grained/hybrid approaches can be applied during the sampling and scoring steps for modeling biomolecular interactions and end with a short discussion of backmapping approaches to restore full atomistic representations.

### 3.1 Simplified representations and topologies

In general, a coarse-grained model aims at decreasing the complexity of a system by grouping several atoms into larger “pseudo-atoms” or “beads”, thereby reducing the number of degrees of freedom. This results both in more efficient computations and a possible smoothening of the energy landscape that might facilitate the identification of relevant states of the system. In the context of proteins, the simplest models introduced are the hydrophobic/polar (HP) models (see Fig. 1). These simplify the representations of a polypeptide chain [14] by considering only two type of beads (H and P), which, to some extent, are an approximation of two types of residues: hydrophobic (H) and polar (P) [15]. Albeit very minimalistic, HP representations have proven useful to study larger conformational changes and longer time scales. These models, and their variants, have been extensively studied in the past decade [16], [17], [18], [19] and reviewed elsewhere [20]. Another example of a low-resolution model to represent proteins is SICHO (Side CHain Only) [21]. In the model developed by Kolinski and Skolnick [21], each amino acid is represented as a unique interaction site, located at the center of the side-chain. It is thus computationally very efficient but completely neglects backbone conformations (φ/ψ dihedrals) [22].

In order to overcome the inaccuracies of very simplistic representations, higher resolution models have been developed. PRIMO/PRIMONA, for proteins and nucleic acids, was proposed as a reduced quasi-atomistic resolution model [23]. Feig and co-workers [23] represent polypeptide backbones with three beads (Cα, N and a combined carbonyl site) and side-chains as a combination of up to five different particles. In the case of nucleotides, adenine, cytosine and uracil are represented by four coarse-grained particles, and guanine and thymine by five. The sugar-phosphate backbone of the PRIMONA model consist of eight different CG beads. In contrast, the HiRE-RNA model designed by Pasquali and Derremaux [24] only considers three of the seven backbone torsional angles (α, β and γ); each RNA nucleotide is represented by six (pyrimidine bases) or seven (purine bases) beads, allowing for a reduction of ~70% of the number of particles compared to a fully atomistic structure. Similar to PRIMO, in the SIRAH model [25] the positions of the nitrogen, α carbon and oxygen from the peptide bonds are kept at pseudo-atomistic resolution, while side chains are treated at a lower degree of detail (from one to five different beads). This model also allows for the study of protein-DNA interactions by molecular dynamics through the use of an explicit/CG solvation scheme [26], [27].

Other coarse-grained models have been designed to be easily transferable and applicable to multiple systems. Among those, MARTINI is probably the most popular one. The current “*MARTINIdome*” includes: lipids [28], proteins [29], polymers [30], [31], carbohydrate [32], water [33], glycolipids [34], nucleotides [35], [36] and nanoparticles [37]. The systems are represented by four different basic particles – nonpolar (N), polar (P), apolar (C) and charged (Q) – that are further classified based on their degree of polarity and hydrogen bonding properties, giving a total of eighteen unique “building blocks”. The MARTINI force field for proteins, in its latest official release (2.2p), includes off-center charges for polar and charged residues [38]. These represent a good proxy for hydrogen bond and salt bridges formation and thus for molecular recognition. For nucleic acids, much like PRIMONA, the MARTINI model specifically accounts for Watson-Crick base pairing (eight additional beads) to stabilize the DNA double helix structure.

### 3.2 Parametrization of coarse-grained force fields

#### 3.2.1 Classical parametrization strategies

In the context of molecular modeling, the set of parameters and functions used to calculate the potential energy of a system is commonly referred to as force field. Atomistic force fields provide parameters usually for every type of atom in a system (hydrogen included) but also united atom representations are often used in which non-polar hydrogens are neglected. In contrast, coarse-grained potentials are a cruder representation of the inter- and intra-molecular interactions. Regarding the latter, their parametrization follows two main routes: Hierarchical (*bottom-up*) and pragmatic (*top-down*) coarse-graining [39].

The key idea of hierarchical coarse-graining is that, the interactions at a less detailed level are the result of the collective interactions at the more detailed level [40]. As an illustration, in the 1975 abovementioned study by Levitt and Warshel [8], the interactions between coarse-grained sites were derived in a *bottom-up* way by explicitly summing up all microscopic interactions of an atomistic model. One obvious limitation of these models is that the quality of the coarse-grained model highly depends on the accuracy of the underlying atomistic one. Similarly, the seminal force-matching (FM) method proposed by Ercolessi and Adams [41] and further developed by Voth and co-workers [42], [43] under the name of MS-CG (multiscale coarse-graining) uses atomistic-level interactions to derive coarse-grained potentials. In short, those potentials are systematically fitted to atomistic forces by minimizing the mean-square errors between them. Much like iterative Boltzmann (IB) derived models [44], these force fields are usually more accurate as compared to more generic ones. However, they are typically less transferable and require more parametrization effort. These methods, and their extensions [45], have been recently applied to coarse-grained models for proteins such as the UNRES model [46].

Pragmatic force fields, however, are designed in such a way that they reproduce a given chosen (experimental) property [47]. The earlier lattice models (such as HP) represent a well-studied example of *top-down* coarse-graining. These models are typically cheaper to parametrize, easily transferable (to similar systems) and use rather simple analytical potentials [48]. In a similar way and as shown in Fig. 1, methodologies based on reproducing thermodynamical properties have been extensively applied in different branches of chemistry such as physical and organic chemistry. Equations of State (EoS), which are mathematical relationships between the thermodynamic variables of a given system, have been shown appropriate to accurately link the macroscopic properties of the system and the force field parameters [49]. As an example, the powerful SAFT-γ EoS, a variation of the Statistical Associating Fluid Theory (SAFT), has been used to estimate the coarse-grained potentials of the Mie force field [50]. This force field has been recently used to calculate solvation free energies of aromatic compounds, which are broadly used in the pharmaceutical industry for drug design purposes [51].

#### 3.2.2 Machine learning-based parametrization

Machine learning, and especially deep learning, is revolutionizing in the last years many areas of science and technology. Certainly, the most significant breakthrough of the decade in the field of protein folding has been the development of AlphaFold [52]. DeepMind, an artificial intelligence company affiliated to Google, has designed a deep learning-based method that represents a substantial advance as compared to classical modeling techniques [53], [54]. These machine learning methods have been also applied in the development of force fields and are usually purely based on existing data. A general approach to design a machine (deep) learning-based force field typically includes: The generation of reference atomic configurations and forces (QM calculations), the identification of specific signatures, the selection of training and test datasets, the mapping of selected signatures to forces using specific algorithms and the assessment of the resulting predictive model [55]. Deep neural networks [56], adversarial machine learning models [57] and genetic algorithm [58] have been recently shown appropriate for the development coarse-grained force fields. Altogether, machine learning-based parametrization methodologies represent an emerging trend to automatize analytical model building from more complex data, which can deliver faster and perhaps more accurate results with minimal human intervention.

### 3.3 Combining different levels of resolution

An exhaustive, yet accurate, sampling of the conformational landscape is crucial in attempts to model biomolecular interactions and evaluate the underlying energetics. The use of simplified representations offers an effective way of sampling the landscape. However, the reduced accuracy due to the inherent simplifications still limits the systems and processes that can be studied by CG approaches. Hybrid approaches, which typically couple coarse-grained and atomistic-level representations, aim to overcome these limitations by combining different levels of resolution [59]. These combined approaches might be very helpful for quantitative studies (e.g. free energy calculations of large systems [60], [61]), while still reducing the computational cost. They are also particularly useful to include components of a system for which no or only low-resolution structural data are available. A key challenge in hybrid modeling is to integrate the different levels of resolution and to describe the AA/CG interactions. Standard mixing rules [62] have been historically very successful for this task. In short, Lennard-Jones and electrostatic interactions for mixed systems can be averaged and combined with an optimal scaling parameter depending on the size of the system [63]. Besides energetics, it still remains unclear how the interaction between two atoms might be affected by a coarse-grained surrounding as compared to its “native” environment and vice versa [64].

There are several hybrid schemes proposed in the literature, with MARTINI as a popular choice for the coarse-grained representation. One example is the PACE force field [65], [66], which pairs MARTINI (water and lipids) with a united-atom protein model. In this case, the AA/CG parameters are optimized against specific thermodynamic data, which somehow limits its direct applicability to other systems. GROMOS/MARTINI coupling [64] has also been described as a potential alternative. In this work, cross-resolution interactions are calculated via virtual interactions sites on relevant atomistic groups and the standard CG beads, an approach that might lead to unbalanced electrostatics behaviors. For this reason, Wasenaar and coworkers [67] introduced an explicit electrostatic AA/CG coupling on the coarse-grained side. More recently, the CHARMM/PRIMO coupling has been proposed for single hybrid simulation purposes [68]. In the model proposed by Kar and Feig [68], the atomistic segment of the hybrid model was found to structurally deviate more than its corresponding one in a full atomistic model. This suggests that proper mixing of resolutions remains a difficult problem.

In the context of integrative modeling, the integration of experimental data at the various possible levels might have a crucial role for hybrid representations of the system. At the sampling level, data can be used to narrow the conformational search so that binding incompetent and/or irrelevant regions are discarded *a priori*. This strategy has been shown to be best suited compared to post-simulation filtering approaches. It not only outperforms the scenario where data is solely used to discard models with a high degree of uncertainty, but also reduces significantly the computational cost [69]. Data can be also incorporated at the scoring level via a numerical penalty term or as restraining energy potential [70]. As an example, in HADDOCK [71] the distance restraints are incorporated into the scoring scheme via a soft-harmonic potential where the potential becomes linear for violations longer than 2 Å [72], effectively avoiding large forces for high restraints violations. Therefore, the incorporation of data in the modeling might work as a firewall and somewhat reduce the impact of inaccuracies of hybrid schemes in terms of intra- and inter-molecular interactions.

### 3.4 Sampling and scoring schemes

Decreasing the computational cost, as well as the complexity of the system, is a major goal of coarse-grained modeling. By lowering the resolution, the energy landscape becomes smoother and it is therefore, in principle, easier to identify the global minimum. In the context of integrative modeling with HADDOCK, we recently showed that introducing the MARTINI coarse-grained force field results in a substantial increase (8–30%) in the number of near-native models generated [73]. We also find CG sampling schemes in ATTRACT [74], [75], [76] (also hybrid scoring), CABS-dock [77], [78] (also scoring), FRODOCK2.0 [79], InterEvDock2 [80], [81] (also scoring), LZerD [82], [83], MAXDo [84], MCDNA [85] (also scoring), MDockPP [86] and RosettaDock [87] (also scoring in RosettaDock 4.0 [88]). Some of the methods used by these software to sample the conformational landscape includes: Rigid-body energy minimization, Fast Fourier Transformation (FFT) or Molecular Dynamics (Monte Carlo). For the purpose of scoring, coarse-grained molecular dynamics simulations have been also evaluated on a heterogeneous benchmark of protein–protein docking models [89]. Other modeling software such as: DOCK/PIERR [90], GALAXY [91], [92], LightDock [93], MEGADOCK 4.0 [94], [95], PPI3D [96], [97], pyDock [98], [99] and V-D^2^OCK [100] incorporate, to some extent, coarse-grained/hybrid scoring approaches for (quasi)atomistic models.

IMP [101] and PyRy3D (genesilico.pl/pyry3d) are examples of ready-to-use hybrid modeling software for predicting (sampling and scoring) biomolecular assemblies allowing to incorporate experimental data into their calculations. The Integrative Modeling Platform leans on the concept that the resolution of the representation depends on the quantity and quality of the available information. This information is also encoded in a scoring function, whose ultimate goal is to evaluate the uncertainty of the generated models. Andrej Sali and co-workers [2] understand the modeling as an endless cyclic process driven by the continuous acquisition of data. In IMP, the different subunits are represented as a combination of spherical beads of varying sizes (different levels of coarseness). The same subunits can be also be represented as 3D Gaussians (for EM map fitting) and thus combine different resolution scales simultaneously [102]. During the conformational sampling, the relative distances from all the CG beads and Gaussians are either constrained (in rigid bodies) or restrained (in flexible bodies) by the sequence connectivity. For very high degrees of coarse-graining, only geometric considerations, e.g. exclude volume, might be used in the computations. PyRy3D allows for building low-resolution models of large macromolecular assemblies. In the software developed by Kasprzak and Bujnicki (genesilico.pl/pyry3d), proteins and nucleic acids can be represented as rigid-bodies or as flexible shapes. A spatial restraints-driven Monte Carlo approach is used to bring the components together followed by an evaluation via a simple scoring function. For a more detailed list of software that allow for building structural models of multi-subunit macromolecular complexes refer to Table 1.

**Table 1:** Available software for building structural models of protein, peptide and/or DNA complexes that incorporates a coarse-grained/hybrid approach into their protocols. Most of the listed software are available as webserver and/or standalone package.

Modeling platform	System(s)	Characteristics	Link	Reference(s)
ATTRACT	Protein, peptide and DNA	CG sampling and hybrid scoring	attract.ph.tum.de	[74], [75], [76]
CABS-dock	Peptide	CG sampling and scoring	biocomp.chem.uw.edu.pl	[77], [78]
DOCK/PIERR	Protein	Hybrid scoring	clsbweb.oden.utexas.edu *	[90]
FRODOCK2.0	Protein	3D grid potential maps	frodock.chaconlab.org	[79]
GALAXY	Peptide	Hybrid scoring	galaxy.seoklab.org	[91], [92]
HADDOCK	Protein, peptide and nucleic acids	CG sampling	bianca.science.uu.nl/haddock2.4	[73], [103], [104]
IMP	Protein and DNA	Hybrid sampling and scoring	integrativemodeling.org	[101]
InterEvDock2	Protein	Sampling by FRODOCK2.0 and CG scoring	bioserv.rpbs.univ-paris-diderot.fr/services/InterEvDock2	[80], [81]
LightDock	Protein, peptide and DNA	Hybrid scoring	lightdock.org	[93]
LZerD**	Protein and peptide	3DZD representation and hybrid scoring	kiharalab.org/proteindocking	[82], [83]
MAXDo	Protein	CG sampling	lcqb.upmc.fr/CCDMintseris	[84]
MCDNA	Protein and DNA	CG sampling and scoring	mmb.irbbarcelona.org/MCDNA	[85]
MDockPP	Protein	CG sampling	zoulab.dalton.missouri.edu	[86]
MEGADOCK 4.0	Protein	Hybrid scoring	bi.cs.titech.ac.jp	[94], [95]
PPI3D	Protein	Voronoi tessellation-based scoring	bioinformatics.ibt.lt/ppi3d	[96], [97]
pyDock	Protein	CG scoring	life.bsc.es/pid/pydockweb	[98], [99]
PyRy3D	Protein and DNA	Hybrid sampling and scoring	genesilico.pl/pyry3d	–
RosettaDock	Protein	CG sampling and scoring	rosettacommons.org	[87], [88]
V-D^2^OCK	Protein	CG scoring	bioinsilico.org/cgi-bin/VD2OCK/	[100]

* Submission to DOCK/PIERR webserver is no longer supported.

** LZerD has an specific protocol for modeling unstructured protein–protein interactions [83].

### 3.5 Backmapping from coarse-grained to atomistic resolution

The inherent loss of accuracy of coarser representations is a limiting factor when analyzing integrative models of biomolecular complexes. Atomic details, such as specific contacts, are usually essential to understand molecular recognition and it is therefore crucial to accurately reconstruct atomistic models from their CG counterparts [105]. This process is commonly referred in the literature as reverse transformation, inverse mapping or backmapping. There is currently a number of different backmapping protocols proposed, which mostly follow two different stages: (1) The generation of an atomistic structure based on the coarse-grained coordinates, and (2) a relaxation step of the generated AA structure.

For the first step, geometrical interpolation [23], [106], [107], random placement [108] and fragment-based methods [87], [109], [110], [111] are the most used ones. All these methods perform sufficiently well according to backbone deviations (<1.0 Å in general) but side chain reconstruction seems more problematic [112]. Side chain optimization has been extensively studied as it directly applies for protein designing purposes. The most successful methods discretize possible side chain conformations into rotamers and usually require of an exhaustive search algorithm (e.g. Monte Carlo, simulated annealing…) and an effective scoring function for selecting the proper side chain conformation. The backmapped atomistic structures can then be further improved by energy minimization [73], [113] and/or more sophisticated molecular dynamics-based approaches [114]. In HADDOCK, the CG generated models are converted into atomistic resolution by using distance restraints between the atoms and their corresponding coarse-grained beads. Using those restraints, the all-atom models of the individual components of a complex are morphed onto the coarse grained complex by a series of energy minimizations and Cartesian molecular dynamics [73].

## 4 Application examples of integrative modeling of protein interactions

Ultimately, the true value of any biomolecular model is in the structural information and insights that it provides. When speaking about integrative modeling here, we refer to the branch of structural biology whose aim is to gain structural insights into biomolecular complexes by integrating a wide variety of experimental information into computational calculations. There are various challenges associated with the incorporation and use of that information for the modeling of assemblies. However, a detailed overview of those is beyond the scope of this manuscript and have been reviewed in depth elsewhere [115], [116], [117]. The relevance of integrative models is underscored by the fact that the Protein Data Bank [118], [119] has now started to collect them in a new integrative model database (PDB-dev; pdb-dev.wwpdb.org) [120], [121], which ultimately should be merged into the current PDB database. Since 2014, it is possible to archive structural models obtained by combining traditional structural experimental techniques such as NMR spectroscopy, electron microscopy (3DEM), small angle scattering (SAS), atomic force microscopy (AFM), chemical cross-linking, Förster resonance energy transfer (FRET), electron paramagnetic resonance (EPR), mass spectrometry (MS), Hydrogen/Deuterium exchange (HDX) and various bioinformatic approaches, with computational methods. In this section we highlight several examples of integrative structures of protein complexes that have been determined by combining coarse-grained/hybrid computational approaches with experimental information.

Among all archived structures, we find a number of them determined by coarse-grained/hybrid computational methods in combination with a wide variety of structural data (see Fig. 2). Integrative structures derived from chemical cross-linking data are by far the most abundant ones, including models of the heptameric module of NPC [122], the exosome complex [123], the Complement C3(H2O) [124], the E6AP/UBE3A-p53 enzyme-substrate complex [125], Pol II(G) [126], the Proteasome-Ecm29 complex [127] and the canonical/non-canonical human COP9 Signalosome [128]. Protein cross-links have been also combined with other types of experimental information such as three/two-dimensional Electron Microscopy (2DEM/3DEM) and/or SAS to determine structures like the yeast Mediator complex [129] or the native BBSome [130]. Other sources of information such as mutagenesis and NMR data [131] and single molecule FRET data [132] have been also used.

There are also multiple examples of integrative structures, not deposited in the PDB-dev database, which have been modelled by integrative coarse-graining methods. One of those is the ATP synthase membrane motor. Leone and Faraldo-Gómez [133] proposed a computational integrative model based on chemical cross-links, a cryo-EM map (~7Å of resolution) and evolutionary couplings. The initial homology models of either subunits were refined against the experimentally determined cryo-EM map using Rosetta, which starts its conformational exploration in coarse-grained resolution. The computationally generated models were further validated with co-evolutionary and cross-linking data and revealed important mechanistic insights into the function of the ATP synthase. Another representative example is the ISWI ATPase complex. Using upper bound distance restraints based on BS^3^, BS^2^G and UV cross-links, Harrer and coworkers [134] modelled the complex with ATTRACT, which performs a rigid-body energy minimization driven by a coarse-grained force field [109] and the distance restraints provided. The top scoring ISWI models were validated against SAXS data.

The Nuclear Pore Complex (NPC) is probably the largest protein assembly determined by an integrative structural approach to date. It constitutes an eight-fold symmetrical cylindrical complex of 552 copies of 32 different nucleoporin proteins (Nups) [135]. With respect to the computational modeling, the NPC was represented in a multiscale fashion including multiple levels of coarseness. As an illustration, all rigid bodies derived from X-ray, NMR and integrative structures were coarse-grained into two different resolutions. They either mapped single residues or consecutive portions of up to ten different amino acids into larger beads. The modeling was performed using the integrative modeling platform software (IMP) (integrativemodeling.org) [101]. The experimental information available included chemical cross-links, a cryo-ET density map, immuno-electron microscopy localizations, excluded volume, sequence connectivity, the shape of the pore membrane, symmetry and SAXS data, which were used to benefit the sampling, to improve the scoring, to filter out inconsistent models and/or validation purposes. By putting all these data together, they were able to fully describe, at sub-nanometer precision, the structure of the entire NPC.

## 5 Concluding remarks

Over the past decades, coarse-grained/hybrid modeling has been demonstrated as a powerful approach to model biomolecules and their interactions. It extends the capabilities of traditional atomistic protocols. There are multiple models to simplify the three-dimensional representation of biomolecules, each of those specifically designed to answer a specific research question. The choice between different representations directly affects the sampling and scoring capabilities of current modeling approaches. In other words, the smaller the number of pseudo-atoms or beads, the higher the increase in speed but the lower the accuracy of the resulting models. For cases where higher level of resolution is required, multiscale/hybrid modeling might help to alleviate the inherent loss of accuracy of pure coarse-grained models as demonstrated, for instance, in the modeling of the nuclear pore complex. Nevertheless, there is still an urgent need for improving interaction schemes. Coarse-grained force fields derived from classical molecular mechanics are not easily transferable and therefore, very much system-dependent. On the contrary to *bottom-up* strategies, *top-down* approaches aim to generalize structural patterns that have been seen in thousands of known structures and/or to reproduce thermodynamic quantities. Likely, a combination of *bottom-up* and *top-*down approaches is a better option. In other words, improving *top-down* models by inferring additional interaction terms derived by *bottom-up* coarse-graining might have the most impact in future designs, increasing both their accuracy and applicability range to wider, larger and more complex assemblies. We are now approaching a time where, taking advantage of all scientific and technological advances, one might expect to build reasonable three-dimensional models of cells, which might provide insights into still unknown cellular mechanisms.

## Declaration of Competing Interest

The authors declare that they have no known competing financial interests or personal relationships that could have appeared to influence the work reported in this paper.
